# Hospital-Based Food Environment Interventions to Improve Workforce Dietary Behaviour: A Systematic Literature Review

**DOI:** 10.1177/15598276231184813

**Published:** 2023-07-04

**Authors:** Kawther al-Tamimi, Louise van Herwerden, Malika Abdul, Jennifer Utter

**Affiliations:** 1 Faculty of Health Sciences and Medicine, 3555 Bond University, Robina, QLD, Australia; 2 Dietetics and Food Services, 3555 Mater Health, South Brisbane, QLD, Australia

**Keywords:** public health, hospital staff, environmental nutrition, hospital food environment

## Abstract

The aim of this study was to determine the effectiveness of hospital-based interventions in improving eating patterns and food purchasing behaviours of hospital staff. Four electronic databases were searched from inception to November 2021 for intervention studies conducted within hospital retail food environments. Studies assessing outcomes pertaining to changes in eating patterns and/or food purchasing behaviour were included. Intervention effectiveness was defined as a statistically significant change in primary outcomes. Study quality was assessed independently by two researchers using the Mixed-Methods Appraisal Tool. In total, 20 studies were included in the review. 10 studies were moderate to low quality and almost half of the included studies (n = 9) utilised a quantitative descriptive design. Intervention modes included signage and health labels (n = 17), price modifications (n = 9), recipe modifications (n = 7), choice architecture (n = 5), altering healthy food availability (n = 5) and cooking processes (n = 1). Most studies (n = 15) contained more than one intervention type within their intervention design. Significant improvements in all primary outcomes were identified within eight studies, in which all had included a point-of-purchase prompt. Multicomponent interventions that incorporate point-of-purchase prompts may be useful in improving eating patterns and/or food purchasing behaviour of hospital staff. Further rigorous research identifying the long-term effectiveness of these interventions on improving health outcomes is warranted.

‘Regarding education-based environmental interventions, evidence for their effectiveness in improving eating behaviour and/or food purchasing behaviour was largely mixed’.

## Introduction

It is well recognised that diet plays a significant role in reducing the occurrence of non-communicable diseases (NCDs) and maintaining wellbeing.^1-3^ Concurrently, certain industries and occupational groups, such as the healthcare workforce, have been identified to be at a higher risk of developing NCDs than other occupational groups.^4^ This has been attributed to the presence of unique industry factors such as long working hours, shift work and high job stress.^4^ A recent systematic review evaluating the relationship between shift work and eating habits among adult healthcare workers recognised that those engaged in shift work consumed higher quantities of high-fat foods and soft drinks compared to rotating or daytime workers.^5^ Further, a survey conducted among healthcare workers within the United Kingdom highlighted that over half did not meet national guidelines for fruit and vegetable intake and more than one-third consumed foods high in fat and sugar on a daily basis.^6^

Unhealthy lifestyle behaviors, such as consuming an unbalanced diet, not only increases disease risk, it further contributes to higher rates of absenteeism and reduced productivity within the workplace.^7^ In particular, a cross-sectional study highlighted that employees who consumed high-quality diets were less likely to be absent from work.^7^ Such findings may be attributed to a range of personal and environmental factors that happen to influence food choice, such as sleep deprivation, circadian clock misalignment and limited availability of healthy foods within the work environment.^8,9^

Several existing systematic literature reviews have reported on the effectiveness of environmental interventions on improving dietary behaviour.^10-12^ These interventions exist in many forms and have commonly-applied environmental strategies involving increasing accessibility to healthy foods or food labelling.^11^ For example, choice architecture, a technique that describes the way in which decision-making is influenced by how choices are presented, has previously been shown to influence food choice.^11,13^ Similarly, point-of-purchase prompts, which include motivational messages such as posters, front-of-package labels or signs used to encourage or persuade the purchasing of certain products, have also been useful in improving healthy food purchases.^14-16^ Additionally, the application of traffic-light labels such as ‘green’, ‘amber’ and ‘red’ used to indicate the healthfulness of a food or beverage based on the quantity of certain nutrients or presence of ingredients, is another commonly-applied strategy across institutions to promote healthy-eating patterns.^13,17^

The hospital environment is unique in that it is a 24-hour workplace that employs a diverse body of staff across the organisation. However, no reviews have examined the effectiveness of selected environmental interventions on influencing the eating patterns of hospital staff.^16,18^ As well, no review to date has synthesised the effectiveness of such interventions within the hospital setting. As such, the aim of this review is to evaluate the effectiveness of hospital-based interventions on improving the eating patterns and/or food purchasing behaviour of hospital staff. Findings from this research have the potential to identify the most effective strategies to improve the hospital food environment, thus assisting policy makers and hospital management to effectively address the dietary concerns of the healthcare workforce.

## Methods

The current review was conducted in accordance with the Preferred Reporting Items for Systematic Reviews and Meta-Analyses (PRISMA) reporting guidelines.^19^

### Search Strategy

Four electronic databases (PubMed, Embase, CINAHL and Web of Science) were searched from inception to November 2021. The search strategy was refined through consultation with an academic librarian and included search terms relevant to population (hospital staff), intervention (point-of-purchase prompt, choice architecture, price and menu modification) and environment (hospital retail outlet and hospital cafeteria). The complete search strategy for all databases can be found in Supplementary Material 1. Reference lists of included studies were searched to identify published studies that the electronic search did not detect. Once studies were de-duplicated using the systematic review accelerator tool,^20^ references were imported into Covidence^21^ and screened against the inclusion criteria. Studies were included if they were intervention studies that introduced environmental change to a hospital food environment, published between 2000 to 2021 and measured outcomes of either food purchasing behaviour and/or dietary patterns within hospital staff. Restricting the date of publication between 2000-2021 ensured that only relevant articles were retrieved, as the hospital food environment has encountered a range of changes since the 19th century.

### Selection Process

Title and abstract screening was completed independently by two trained researchers. Any disagreements were managed by discussion and, where necessary, by a third member of the research team. Title and abstract screening was followed by the retrieval and screening of full-text articles, where two authors independently screened all studies according to the eligibility criteria.

### Data Synthesis

Microsoft Excel was utilised to extract and synthesise data from the included studies. One reviewer conducted the extraction whilst another reviewer checked the extracted data for accuracy. The extracted data included: author, location, study design, duration and setting, sample size, intervention type, outcomes assessed and measurement of outcomes. To answer the research question, intervention effectiveness was defined by a statistically significant change in primary outcomes (purchasing behaviour and/or diet patterns) whilst also taking into account study limitations. As the included studies varied in terms of study design and intervention type, performing a meta-analysis was not feasible.

### Quality Appraisal

Assessment of quality was conducted using the mixed-methods appraisal tool (MMAT).^22^ Two screening questions and five quality criteria items were used to appraise the methodological quality of the included studies. Each item was rated on a categorical scale with the number of items rated ‘yes’ counted to provide an overall score. Two reviewers independently appraised all included studies with any discrepancies in rating between reviewers resolved through discussion.

## Results

### Overview of Studies

**Figure 1.**
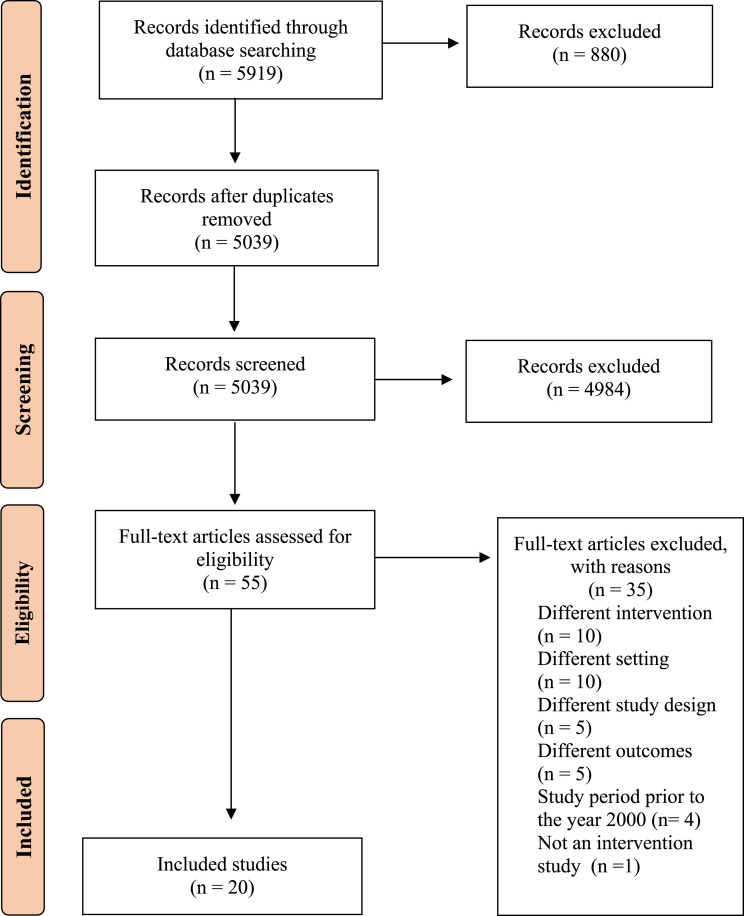
Prisma flow diagram of selected studies.

The initial search yielded 5919 research articles. Figure 1 details the process of identifying the articles for inclusion in this review. Twenty articles met the inclusion criteria and were eligible for data synthesis. Studies were excluded at the screening and full-text review stages if they did not meet the eligibility criteria for intervention type, study design, outcome measures, setting or if the study period was prior to the year 2000. Key characteristics of the included studies are summarised in Table S1. Three were randomised control trials,^23-25^ five were non-randomised control studies,^26-30^ 11 were quantitative descriptive studies^31-41^ and one was mixed-methods.^42^ Overall, 11 studies were conducted in the United States^24-26,28,32-35,37,39,40^ followed by Canada (n = 2),^30,41^ Netherlands (n = 2),^31,38^ Scotland (n = 1),^23^ Australia (n = 1),^42^ Ireland (n = 1),^27^ Denmark (n = 1)^29^ and United Kingdom (n = 1).^36^ Eighteen studies were published within the last 10 years.

### Quality of Included Studies

**Figure 2.**
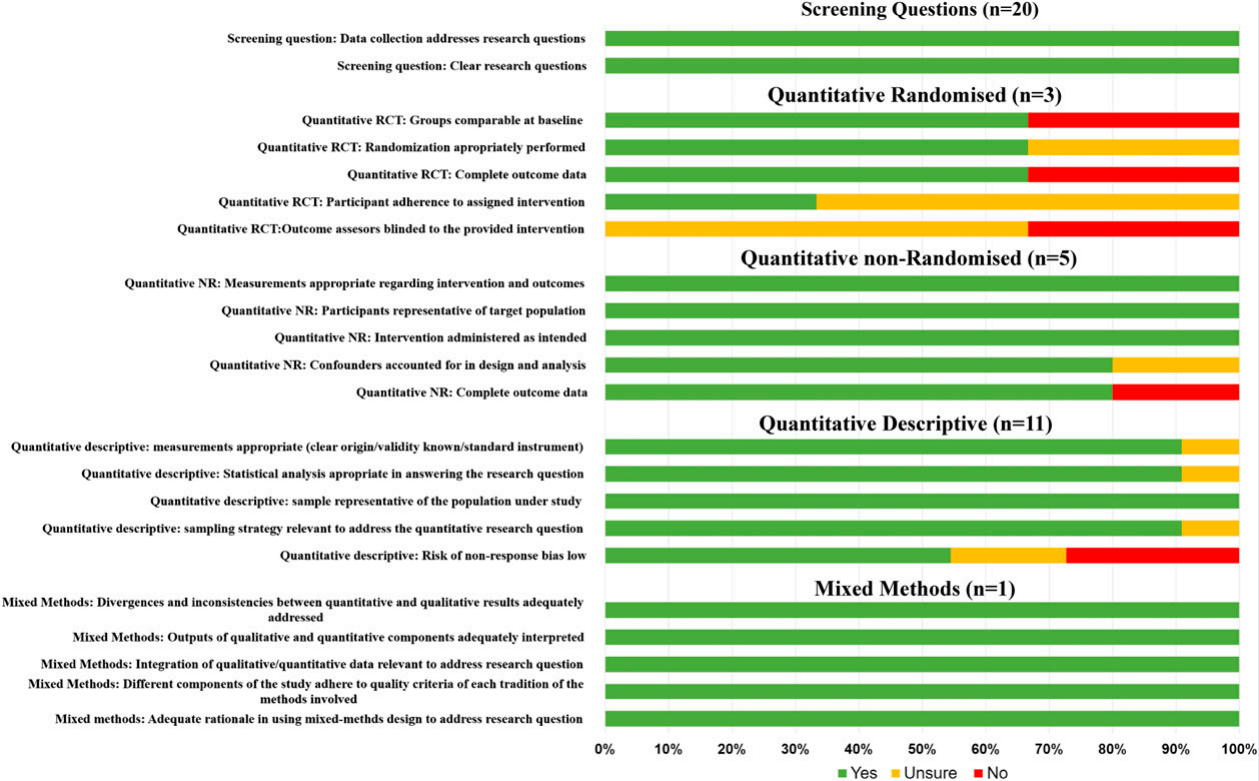
Quality assessment of included studies using the MMAT critical appraisal tool.

Overall, the methodological quality of the studies was mixed (Figure 2). Both the quantitative randomised and quantitative non-randomised studies were given a high-quality score for three out of the five criteria questions. The quantitative descriptive studies were rated as high quality for one of the criteria questions, whereas the mixed-methods study was issued a high-quality rating for all five criteria questions. It should be noted, however, that the MMAT does not report on appropriateness of study design, which was a key limitation of the studies included.

### Intervention Components and Effectiveness

Table 1 displays the different intervention types implemented across the included studies. Point-of-purchase prompts, including health messages/signs and nutrition information, were integrated within 12 studies.^10,23-25,27-31,34,35,39,41^ Six studies integrated health labels^26,28,29,32,35,39^ whilst five integrated traffic-light labels^24,25,28,33,37,42^ within their intervention design. Price modification was assessed within nine studies,^2,4,27,28,30,32,35,39,40,42^ whilst recipe modifications were assessed in seven studies^24,25,29,31,34,39,41^ and choice architecture assessed within five studies.^25,31,33,36,38^ Interventions that increased healthy food availability were incorporated within four studies^28,35,36,41^ and the removal and/or restriction of unhealthy foods was performed within three.^34,36,41^ Lastly, only one intervention observed changing the cooking process within the hospital food environment.^34^ Fifteen studies incorporated more than one intervention type within their design, with four studies incorporating four different intervention types and three studies incorporating five. Table three describes reported intervention effectiveness when measured against primary outcomes. Eight studies observed statistically significant improvements in all outcomes^23,29,30,33,34,37,41,42^ whilst two studies observed statistically insignificant changes on all outcomes pertaining to food purchasing behaviour and/or dietary patterns.^36,39^ The majority (n = 10) noted significant improvements in only a partial quantity of outcomes assessed.^24-28,31,32,35,38,40^

**Table 1.** Intervention Types of Included Studies.

InterventionTypeStudies (n = 20)	Point-of-purchase prompt*				Choice architecture (n = 5)	Price modification (n = 9)	Recipe modification (n = 7)	Increasing healthy food availability (n = 4)	Removal/restriction of unhealthy foods (n = 3)	Change in cooking processes (n = 1)
	Health message/sign (n = 7)	Traffic-light labels (n = 5)	Nutrition information (n = 7)	Health labels (n = 6)						
Allan et al^23^	✓									
Blake et al^42^		✓				✓				
Block et al^26^	✓					✓				
Dawson et al^41^	✓		✓				✓	✓	✓	
Dorresteijn et al^31^	✓				✓		✓			
Elbel et al^32^				✓		✓				
Geaney et al^27^			✓				✓		✓	✓
LaCaille et al^28^	✓	✓	✓		✓		✓			
Lassen et al^29^				✓						
Levy et al^33^		✓			✓					
Lowe et al^24^			✓			✓	✓			
Mazza et al^34^	✓	✓		✓		✓		✓		
Patsch et al^35^	✓			✓		✓		✓		
Sato et al^39^			✓	✓		✓	✓			
Simpson et al^36^					✓			✓	✓	
Sonnenberg et al^37^		✓								
Stities et al^25^			✓			✓				
Vanderlee et al^30^			✓	✓			✓			
Van Kleef et al^39^					✓					
Warsaw & Morales^40^						✓				

### Education-Based Interventions

Education-based interventions (n = 17) were those that incorporated health messages, health and/or traffic-light labels or nutrition information at the point of purchase. Four studies assessed the effect of education-based interventions in isolation,^23,26,29,37^ and the remaining (n = 13) integrated additional intervention components and analysed the combined intervention effect on primary outcomes. Overall, two out of the four studies that assessed education-based interventions in isolation identified significant improvements in purchasing behaviour and/or dietary patterns (Table S2).^23,26,37^ Although the remaining study highlighted partial improvements in dietary measures when implementing nutrition information, a significant reduction in fruit and vegetable consumption was also observed.^29^

### Choice Architecture

Five studies incorporated choice architecture within their intervention design, in which two recognised significant changes in primary outcomes assessed,^31,33^ two delineated partial improvements^25,38^ and one identified no improvement in dietary patterns nor food purchasing behaviour.^36^ Three studies assessed the effect of choice architecture independently^31,33,38^ whilst two integrated additional intervention components and analysed the combined intervention effect on primary outcomes.^25,36^ Two out of the three studies that assessed the effectiveness of choice architecture independently identified an improvement in purchasing behaviour, where one noted a reduction in the purchase of ‘red’ beverages relative to the preceding traffic-light labelling phase.^33^ However, results from van Kleef and colleagues^38^ described that although a shelf assortment of 75% healthy vs 25% unhealthy increased daily healthy snack sales, the snack manipulations did not impact sales of unhealthy snacks on offer.^38^

### Price Modifications

Nine studies incorporated price modification to food and/or beverage items within their intervention design in which two observed significant changes in all primary outcomes^23,34^ which included items purchased and nutritional content of purchased items. Five highlighted partially significant changes^24,27,32,35,40^ and one study identified no significant change on food purchasing behaviour nor dietary patterns.^28^ Out of the five studies that assessed the effect of price modifications independently, two highlighted significant improvements in consumer purchasing behaviour of unhealthy beverages, sugar and total energy,^27,32^ whilst one identified no significant impact.^28^ Across all studies, the percentage price modification ranged between 20-35%.

### Alterations in Availability of Healthy and Unhealthy Foods and Beverages

Ten studies incorporated interventions that altered the foods and beverages offered within the hospital cafeteria to enhance the proportion of healthy items available. All 10 studies incorporated additional intervention components within their design, where three noted significant improvements in dietary patterns and/or food purchasing behaviour,^29,34,41^ four outlined partial improvements^24,25,31,35^ and three identified no improvement in outcome measures^28,36,39^ (Table 1). The three studies that indicated significant improvements had combined an education-based intervention (nutrition information) within their design.

### Multicomponent Interventions

Of the 20 included studies, 15 incorporated more than one intervention type within their intervention design. All 15 studies integrated an education-based intervention within their intervention design, whilst eight included a price modification to food and/or beverage products. Overall, five identified significant improvements in eating patterns and/or food purchasing behaviour,^25,27,29,30,33,34,41,42^ seven recognised partial improvements^24,26,28,31,32,34,35^ and two noted no improvements on outcomes measured (Table S2).^35,36^ Although one study identified improvements in staff eating patterns after the implementation of a workplace cafeteria program, it is undetermined if the changes yielded statistical significance.^41^

## Discussion

The aim of the current systematic review was to evaluate the effectiveness of hospital-based interventions on improving the eating patterns and/or food purchasing behaviour of hospital staff. Key findings from this review suggest that changes to the hospital food environment can have a positive impact on the nutrition of healthcare workers where the most successful interventions are those that include more than one environmental change. This is consistent with findings from previous research, where Roy and colleagues^43^ highlighted that a combination of interventions was more effective than a single intervention in improving eating behaviors of adults employed in tertiary education settings.

The effectiveness of single environment-based interventions, which include point-of-purchase messaging, choice architecture and increasing the availability and/or accessibility of healthier foods, was mixed. However, a noteworthy finding from the eight studies that identified significant improvements on all primary outcomes assessed was that each had incorporated a point-of-purchase prompt such as traffic-light labelling, nutrition labelling or health messaging within their intervention design. Reported effects included: reduction in total energy,^23,29,30^ fat,^30,34^ added sugars^26,34^ and sodium^29,34^ content of food purchases and positive changes in the purchase of ‘red’ and ‘green’-labelled items.^33,42^ Although three out of the 10 highest quality studies delineated significant improvements in eating patterns and food purchasing behaviour after implementing interventions that included a point-of-purchase prompt, the remaining six that incorporated a point-of-purchase prompt identified partial improvements with notable effect. For example, Block and colleagues^26^ identified that although no significant change in water purchases were observed after the implementation of a price incentive and point-of-purchase prompt, significant reductions in soft drink sales were recognised.^27^ As such, it becomes apparent that the inclusion of a point-of-purchase prompt with an additional intervention, such as choice architecture or price modification, may be a useful tool in influencing eating patterns and/or purchasing behaviour of hospital staff. This outcome is consistent with findings from previous reviews that identified combining interventions such as point-of-purchase prompts with a pricing, promotional and advertising component to be the most effective in promoting healthier food choices and eating practices in supermarket and grocery stores.^44,45^

Regarding education-based environmental interventions, evidence for their effectiveness in improving eating behaviour and/or food purchasing behaviour was largely mixed. Substantial positive changes pertaining to energy content of food purchases, sales of regular soft drinks and consumption of wholegrains was identified in some, but not all studies.^29,37,41^ Although the evidence regarding the provision of nutrition information is conflicting, a systematic review conducted by Fernandes and colleagues^46^ highlighted that menu labelling more frequently presented a partial influence on food choice as opposed to an overall or no influence. The authors further concluded that traffic-light labels were the most effective strategy in promoting healthy eating behaviour.^46^ As only one study within this review applied traffic-light labels on their own,^37^ in which statistically significant changes in food purchasing behaviour were observed, the challenge remains in identifying the absolute effectiveness of traffic light labelling within a hospital setting.

Similarly, the effectiveness of choice architecture in influencing behaviour change varied between studies. For example, Levy and colleagues^33^ utilised cash register data to highlight the changes in the purchase of ‘red’ and ‘green’ foods once cafeteria items had been rearranged to ensure ‘green’ labelled items were more accessible than ‘red’ labelled items. Although the authors concluded significant changes in food purchasing behaviour, this effect was not completely reflected by van Kleef and colleagues,^38^ who measured shelf arrangement (accessibility) and assortment structure (availability) of snacks within a hospital restaurant. It was concluded that significant changes in purchasing behaviour were observed only during changes in assortment structure, not shelf arrangement.^38^ In other words, increasing the proportion of healthy items was more effective in increasing healthy item sales as opposed to making unhealthy items less accessible. On the other hand, Dorresteijn and colleagues^31^ identified that reversing the accessibility of butter and margarine to make butter the more accessible option increased their purchase. This effect disappeared when the assortment was restored to the baseline condition. As such, choice architecture may encourage healthier food purchases; however, further research with more rigorous study design is required to confirm this. In addition, a recent systematic review on choice architecture interventions confirmed that behaviour change was most successful amongst interventions that involved changes in the availability and proximity of healthy foods, as partially observed in the findings of included studies within this review.^18^

Interventions involving the application of a price incentive and/or disincentive, such as reducing the price of healthy foods and beverages and increasing the price of less-healthy products, produced mixed results. Interestingly, three out of the eight studies that incorporated a price increase highlighted significant changes in the purchase of less-healthy foods^32^ and sugar-sweetened beverages,^27^ and an increase in the purchase of ‘green’ labelled products.^42^ While there is a need for further high-quality research in this area, these results are supported by findings obtained from Sawada and colleagues,^44^ who highlighted that incentive-based interventions may lead to improvements in fruit intake.

The ability to draw strong conclusions on the effectiveness of environmental interventions on improving dietary patterns and/or food purchasing behaviour remains a challenge. This is due to the presence of various limitations revolving around methodological weaknesses such as inappropriately matched control groups, small sample sizes and the absence of long-term follow-up. Six studies measured changes in primary outcomes using self-reported data collection, either through surveys, diet recalls or interviews. This makes it probable that reporting bias occurred, as according to van Assema and colleagues,^47^ individuals have a tendency to overestimate fruit and vegetable intake whilst underestimating fat intake.

To our knowledge, this is the first systematic review to focus exclusively on environmental interventions targeted at improving eating patterns and food purchasing behaviour of hospital staff. The comprehensive assessment of the quality and reporting of the included studies as well as the classification of studies into specific intervention types are key strengths of this review. However, the heterogeneity of the included studies is the primary limitation of this review, in which outcome measures limited data pooling and the ability to perform a meta-analysis. Further, methods utilised to assess changes in diet quality were not consistent across all studies. Questionnaires, sales data and interviews were some of the key methods used in which some of the questionnaires used involved the use of either a food frequency questionnaire or 24 hour recall. Although both methods are common tools for collecting nutritional data, not all studies implemented the use of validated questionnaires within their study design. Finally, the MMAT tool used to quality appraise the included studies does not report on appropriateness of study design, which was a key limitation of the studies included. Future research with more rigorous experimental design is needed to assess the influence of food-environment changes on diet quality within this population group. As well, additional research comparing the effectiveness of individual and multicomponent interventions is needed.

## Conclusion

In conclusion, a range of single and multi-component interventions have been developed with the aim to improve the healthfulness of the hospital food environment for staff. The adoption of interventions such as choice architecture, price incentives and point-of-purchase prompts may be potentially useful in improving eating patterns and/or food purchasing behaviour of hospital staff; however, more high-quality research is required to confirm this. Finally, the implementation of a point-of-purchase prompt combined with an additional intervention was largely identified as having a positive effect on staff purchasing behaviour.

**CME/CE Article Quiz**

American College of Lifestyle Medicine (ACLM) members can earn FREE CME/CE credit by reading this approved CME/CE article and successfully completing the online CME/CE activity. Non-members can earn CME/CE for $40 per article. Visit lifestylemedicine.org to join the ACLM.

**Instructions.**

1. AJLM CME/CE Articles and Quizzes are offered online only through the American College of Lifestyle Medicine and are accessible at lifestylemedicine.org/store. ACLM Members can enroll in the activity, complete the quiz, and earn this CME/CE for free. Non-members will be charged $40 per article.
2. A Passing score of 80% or higher is required in order to be awarded the CME/CE credit.

## Supplemental Material

Supplemental Material - Hospital-Based Food Environment Interventions to Improve Workforce Dietary Behaviour: A Systematic Literature Review Supplemental Material for Hospital-Based Food Environment Interventions to Improve Workforce Dietary Behaviour: A Systematic Literature Review by Kawther al-Tamimi, Louise van Herwerden, Malika Abdul and Jennifer Utter in American Journal of Lifestyle Medicine
