# Supplementary material for: Hospital-Based Food Environment Interventions to Improve Workforce Dietary Behaviour: A Systematic Literature Review
Source: Am J Lifestyle Med. 2023 Jul 4;19(4):616–25. doi: 10.1177/15598276231184813 (PMC12000846; doi:10.1177/15598276231184813)
Supplement: Supplemental Material - Hospital-Based Food Environment Interventions to Improve Workforce Dietary Behaviour: A Systematic Literature Review [file sj-pdf-1-ajl-10.1177_15598276231184813.pdf]

Supplementary material 1: Search Strategy

Search Strategy

(hospital[Title/Abstract] OR "Hospitals"[MeSH Terms] OR "workplace"[MeSH Terms] OR "Food Service, Hospital"[MeSH Terms] OR "Hospitals, University"[MeSH Terms])

AND

("Humans"[MeSH Terms] OR "Adult"[MeSH Terms] OR "Young Adult"[MeSH Terms] OR "Adolescent"[MeSH Terms] OR "Male"[MeSH Terms] OR "Female"[MeSH Terms] OR "Health Personnel"[MeSH Terms] OR "healthcare staff"[Title/Abstract] OR employee[Title/Abstract] OR “hospital staff”[Title/Abstract])

AND

("Diet, Healthy"[MeSH Terms] OR nutrition[Title/Abstract] OR "Feeding Behavior"[MeSH Terms] OR "food purchasing"[Title/Abstract] OR “food purchases”[Title/Abstract] OR "Choice Behavior"[MeSH Terms] OR "Body Mass Index"[MeSH Terms] OR "Energy Intake"[MeSH Terms] OR "Body Weight"[MeSH Terms] OR "Nutritional Status"[MeSH Terms] OR (cafeteria[Title/Abstract] AND purchasing[Title/Abstract]) OR “healthy food choices”[Title/Abstract])

AND

(nudge[Title/Abstract] OR "behavioral intervention"[Title/Abstract] OR "point of purchase"[Title/Abstract] OR "food availability"[Title/Abstract] OR "healthy food"[Title/Abstract] OR "choice architecture"[Title/Abstract] OR "public health policy"[Title/Abstract] OR retail[Title/Abstract] OR vend*[Title/Abstract] OR traffic-light[Title/Abstract] OR "Health Promotion"[MeSH Terms] OR "food environment"[Title/Abstract] OR "Food Labeling"[MeSH Terms] OR "Nutrition Policy"[MeSH Terms] OR "Affect/Physiology"[MeSH Terms] OR incentive[Title/Abstract] OR "Snacks/Classification"[MeSH Terms] OR “food choice”[Title/Abstract] OR point-of-decision[Title/Abstract])

Author, location	Study design, duration and setting	Sample size	Intervention	Outcomes assessed	Time points for assessed outcomes	Measurement of outcomes
**Allan et al[23]**<br><br>**Scotland**	RCT (cluster)<br><br>24 wks<br><br>30 Hospital Cafeterias	Intervention group: 15 cafeterias<br><br>Control group: 15 cafeterias	Point-of-purchase prompt – Intervention site: Sign highlighting dietary energy content of all single-serve snack foods from lowest to highest<br><br>Control site: no point-of-purchase prompt.	**Primary:** Dietary energy content of purchased products<br><br>**Secondary:** Average fat and sugar content of products purchased, average cost of each product purchased and number of products purchased daily.	Baseline: 12-week data collection period pre-intervention installation<br><br>Follow-up: 12-week data collection post intervention installation	Purchasing data collected from the retailer.
**Lowe et al[24]**<br><br>**USA**	RCT<br><br>12 wks<br><br>2 Hospital Cafeterias	Intervention group A: 53 employees<br><br>Intervention group B: 43 employees	Two intervention conditions –<br><br><u>Environmental changes (EC):</u> Reducing dietary energy content of foods within cafeteria and provision of nutritional labels for all foods sold. Healthier food items added without removing existing energy-dense foods. Modification of recipes enabling provision of both original recipe and a healthier alternative.<br><br><u>Environmental changes plus (EC-Plus):</u> Environmental change condition plus training	**Primary**: Food choices<br>**Secondary:** Naturalistic food intake.	Baseline: 2-month period of data collection pre-intervention installation<br><br>Full Intervention: 3 month period of data collection during intervention<br><br>Follow-up: 6-months and 12-months post-intervention installation.	Cafeteria intake: Identification cards swiped at cash register captured and automatically saved ID numbers and unique code associated with all the food items sold. Naturalistic food intake: 24-hour diet recalls

			on how to reduce dietary energy of diet plus discounts (15% off for 'low energy density' and 25% off for 'very low energy density' items).			
**Stities et al[25]** **USA**	RCT<br><br>4 wks<br><br>1 Hospital cafeteria	Intervention group: 10 employees<br><br>Delayed-intervention group: 16 employees	<u>Online pre-ordering system –</u> System listing all cafeteria foods with associated dietary energy content, fat and ingredient information.<br><br>Three study phases - Baseline – Participants selecting lunch meals from cafeteria without the use of pre-ordering system.<br><br>Full intervention - Participants received mindful eating training and offered financial incentives to encourage pre-ordering lunch meals.<br><br>Partial intervention - encouraged to pre-order lunch meals without being offered a financial incentive.	**Primary**: Dietary energy content and fat of lunch purchases	Baseline: 4–8-week data collection period pre-intervention installation<br><br>Full intervention: 4-week period of data collection during intervention<br><br>Partial intervention: 4-week period data collection	Purchasing data obtained from cafeteria point-of-sale system. Dietary energy content and fat based on food service providers meal planning guide. If unavailable, obtained from food manufacturer or online database.

**Blake et al**[42]<br><br>**Australia**	Convergent parallel mixed methods<br><br>17 wks<br><br>1 Hospital convenience store	Survey participants: 352 employees<br><br>Interview participants:<br><br>4 employees.	Traffic-light labelling – Beverages classified as 'green', 'amber' and 'red' using a state government framework.<br><br>Price increase -<br>20% price increase on 'red' beverages	**Primary:** Changes in sales, beverage volume and revenue	Baseline: 172-week period of data collection period pre-intervention installation<br><br>Full intervention: 17-week period of data collection during intervention	Sales data collected from cash register.
**Dawson**[41]<br><br>**Canada**	Descriptive Study<br><br>4 wks<br><br>1 Hospital cafeteria	Intervention group: 504 employees (51% response rate)	Multi-component intervention testing five difference point-of-sale interventions to promote healthy eating behaviour. Interventions included:<br><br>1. Point-of-purchase prompt (signs highlighting different nutrition messages, healthy food options and nutrition value of certain menu items)<br>2. Food demonstration with free samples<br>3. Free apples in the cafeteria check-out line<br>4. Condiments made available on request<br>5. Encouragement of cream/<br>6. vegetable soup or salad to accompany sandwich at a	**Primary:** Frequency of visits to and purchases made within hospital cafeteria<br><br>Short-term eating behaviour change	Not available.	Questionnaire – Frequency of visits and purchases assessed through 3 close-ended and 2 open-ended questions.<br><br>Self-reported eating behaviour change measured through 2-close ended questions.

			special price.			
**Dorresteijn et al[31]**<br><br>**Netherlands**	Single-blind uninterrupted time-series<br><br>8 wks<br><br>1 Hospital cafeteria	Mean daily number of cafeteria visitors/day during weekdays at:<br><br>Baseline - 2273<br><br>Intervention period - 2193<br><br>Post-intervention - 2250	Point-of-purchase prompt – Signs promoting reduced-salt soup and reduced fat croissant.<br><br>Choice architecture – Reversal of the availability and accessibility of diet margarine and butter (butter made more accessible).	**Primary:** Number and ratio of normal and reduced-salt soup purchased<br><br>Full-fat butter and lean croissants purchased<br><br>Diet margarine and butter cups purchased	Baseline: Daily 2-week period of data collection period pre- intervention installation<br><br>Full intervention: Daily 2-week period of data collection during intervention<br><br>Post-intervention: Daily 2-week period of data collection post-intervention installation.	Sales data collected from computer system at pay desk for soups, margarine and butter purchases.<br><br>Remaining croissants counted and classified as unsold.
**Elbel et al[32]**<br><br>**USA**	Controlled field experiment<br><br>5.9 wks<br><br>1 Hospital connivence store	Total purchases: 3680	Taxation and labelling intervention on foods and beverages.<br>Five study phases –<br><br>1. Baseline<br>2. highlighting the words 'less healthy' in red on the price-tag of foods<br>3. 30% price increase on unhealthy items 4) combination of taxation and labelling<br>4. 30% tax on unhealthy items and reasons for taxation described on label.	**Primary:** Consumer purchasing behaviour	Not available.	Observations of all food and beverage purchases made within the store defined as either:<br><br>1. Healthy item<br>2. Less healthy item<br>3. Healthy beverage<br>4. 4) Less healthy beverage

			5. All intervention phases lasted for 8-9 days.			
**Geaney et al**[27]<br><br>**Ireland**	Cross-sectional comparison study<br><br>12 wks<br><br>2 Hospital cafeterias	Intervention group: 50 employees<br><br>Control group: 50 employees	Multi-component intervention testing impact of catering initiative.<br>Interventions included:<br>1. menu modification and product alternatives to reduce salt content of meals<br>2. provision of salad and vegetables with no extra cost<br>3. removal of salt from all cooking processes (sachets available at service) and introducing herbs and spices<br>4. limiting cooking methods with oil and offering fruit-based desserts only<br>5. 5) display of nutrition information on salt reduction and healthy diet throughout cafeteria.	Primary: Salt and dietary intake	Performed daily during full-intervention phase.	Questionnaires and 24-hour diet recall

**Block et al**[26]<br><br>**USA**	Quantitative non-randomised<br><br>18 wks<br><br>2 Hospital cafeterias and 1 beverage cart	Not available.<br><br>Post-intervention survey: 118 employees.	Price increase and educational intervention.<br><br>Five study phases -<br>1. Baseline<br>2. 35% price increase on regular soda<br>3. washout<br>4. education campaign (poster and information flyer)<br>5. 35% price increase and education.<br><br>All intervention phases lasted for 4 weeks.	**Primary:** Sales of regular soft drinks, diet soft drinks and water.<br><br>**Secondary:** Sales of other beverages, snack food. desserts, total sales, and revenue.	Baseline: Daily 2-week period of data collection period pre- intervention installation<br><br>Full intervention: Daily 16-week period of data collection during intervention implementation.<br><br>Post intervention implementation: 5-day period of cafeteria customer survey collection two-weeks post intervention implementation.	Sales data collected from daily sales sheets provided by the cafeteria.<br><br>Customer surveys on beverage preferences, factors influencing beverage selection and awareness of interventions implemented.
**LaCaille et al**[28]<br><br>**USA**	Quasi-experimental nonequivalent control group<br><br>52 months<br><br>1 hospital cafeteria and 6 primary care clinics	Intervention group (Hospital cafeteria): 407 employees<br><br>Control group (Primary care clinics): 93 employees	Multi-component intervention testing effectiveness of *Go!, a* worksite obesity prevention program.<br><br>Interventions included:<br>1. Dietary energy content labelling<br>2. Traffic-light labelling<br>3. Signs describing number of steps required to 'burn off' calories of specific food items<br>4. Alterations in dessert location	**Primary:**<br>Changes in dietary behaviour	Baseline: Period of data collection unavailable.<br><br>Full-intervention: Data collection at 6 and 12-months during intervention implementation.	17-item self-reported food frequency questionnaire used to measure dietary behaviour

			5. Introducing highly visible cooler stocking healthy foods<br>6. Reducing serving spoon size<br>7. Increasing availability of 'green' foods<br>8. Reducing cost of half-portions and reducing portion size of specific foods.			
**Lassen et al[29]**<br><br>**Denmark**	Quasi-experimental<br><br>6 wks<br><br>2 Hospital cafeterias	Intervention group: 45 employees<br><br>Control group: 45 employees	Evaluation of the effectiveness of a governmental keyhole certification program.<br><br>Intervention site: Use of Nordic keyhole symbol on healthy food items as part of the healthy labelling certification programme.<br><br>Control site: No implementation of Nordic Keyhole symbol.	**Primary**: Nutrient composition of consumed lunch meals (dietary energy intake, energy density, total fat, salt, wholegrain, refined sugar, fruit and vegetable intake and edible plate waste.	Baseline: Period of data collection unavailable.<br><br>Full intervention: data collection six-weeks from baseline.<br><br>Post-intervention: Data collection six months from end of intervention.	Questionnaire used to determine employee satisfaction developed by researchers.<br><br>Photographs and total plate weight of meals taken before and after they were consumed.
**Levy et al[33]**<br>**USA**	Longitudinal<br><br>39 wks<br><br>1 hospital cafeteria	Intervention group: 4642 employees	Point-of-purchase intervention.<br><br>Three study phases:<br>1. Baseline<br>2. Traffic-light labelling of all food items as 'green', 'amber' or 'red' based on the	**Primary**: Changes in purchases of 'green', 'yellow' and 'red' items.<br><br>**Secondary**: Changes in kilocalorie per	Performed daily during:<br><br>Baseline: Daily 3-month period of data collection period pre- intervention implementation	Cash register data linked to employee platinum cards.

			USDA My Pyramid recommendations<br>3. Choice architecture implemented three-months post labelling designed to increase visibility of 'green' foods and reduce visibility of 'red' foods.	beverage and price per beverage.	Full intervention: Daily 3-month period of data collection during intervention implementation.<br><br>Post intervention implementation: Daily 3-month period of data collection post intervention implementation.	
**Mazza et al[34]**<br><br>**USA**	Field study<br><br>91 wks<br><br>1 hospital cafeteria	Not available	Multi-phase intervention testing effectiveness of food-choice interventions.<br>16 intervention phases - Control conditions constituted phase 1, 2 and 3 and included - 25 cent price increase on regular soft drink, 25 cent price decrease on water and addition of traffic light labels to food items.<br><br>Intervention phases included -<br>Phase 4: emoticons (smiley faces and sad faces) to control conditions.<br>Phase 6: health message to control conditions<br>Phase 8: second health message to the control conditions<br>Phase 10: social norms message to the control conditions<br>Phase 12: healthy substitute to a	**Primary**: percentage daily sales of chips and beverages that were healthy	Full-intervention: Daily 21-month period of data collection during intervention implementation.	Daily register journals uploaded to the hospitals database system. Within dataset, food items were categorised as 'green', 'yellow' and 'red'. Each intervention phase was measured by comparing the intervention to the washout phase directly before it.

			less healthy item to the control conditions.<br>Phase 14: Grouping labelled 'green', 'amber' and 'red' items to the control conditions. Phase 16: Removal of traffic light labels while retaining the price changes on soft drinks and water.<br><br>Washout periods occurred at phases 5, 7, 9, 11, 13 and 15 (return to baseline conditions).			
**Patsch et al**[35]<br><br>**USA**	Non-randomised evaluation<br><br>39 wks<br><br>1 hospital cafeteria and 1 medical centre	2800 hospital employees.	Evaluation of Better Bites programme – Worksite initiative involving introduction of healthy version of an existing, unhealthy food item.<br>Intervention included:<br>1. 35% price reduction of healthy items and 35% price increase on unhealthy items.<br>2. Introduction of Better Bites logo on all food items that met the nutritional criteria<br>3. Signs posted around cafeteria highlighting taste, cost and health benefits of healthier items.<br><br>During baseline, healthy items were introduced without	**Primary:** Change in average weekly sales of healthy vs unhealthy food items<br>**Secondary:** Change in proportion of healthy vs unhealthy items by facility from baseline Changes in average monthly cafeteria gross sales<br><br>Changes in average weekly profits for burgers.	Baseline: 3-month period of data collection pre-intervention implementation.<br><br>Full-intervention: 9-month period of data collection during intervention implementation.	Cafeteria cash register data. Addition of register buttons tracked all purchases of introduced items. Nutrition service managers generated weekly reports and both hospitals provided financial data.

			marketing and pricing components.			
**Sato et al[39]**<br><br>**USA**	Quasi-experimental<br><br>8 wks<br><br>1 hospital cafeteria	Surveys completed: 131	Labelling initiative to include dietary energy, fat and sodium content of 'Healthy picks' entrees and 'regular menu' entrees. Label further included pie-chart representing daily percentage of dietary energy, fat and sodium food contributed to a standard 2,000 KJ diet. 'Healthy picks' entrees were modified to contain:<br><br>1. ≤ 35% calorie from fat<br>2. ≤ 10% calories from saturated fat<br>3. 3) ≤ 1000mg sodium per entrée. One 'healthy pick' entrée and one regular menu entrée (recipe not modified) were available daily.	**Primary:** Changes in receipt sales<br><br>**Secondary:** Consumer noticing behaviour	Lunch sales data Tuesday through Friday during:<br><br>Baseline: 4-week period of data collection pre-intervention implementation<br><br>Intervention period: 8-week period of data collection during intervention implementation.	Lunch sales receipts<br><br>Anonymous survey examining consumer noticing behaviour
**Simpson et al[36]**<br><br>**UK**	Quantitative descriptive<br><br>8 wks	Not available.	Multi-component intervention testing effectiveness of increasing healthy food choices on purchasing behaviour.<br><br>Interventions included:<br>1. Removing large sized chocolate bars, sweets, soft drinks and crisps.	**Primary:** Sales of healthier food options<br><br>**Secondary:** Profit	Baseline: 2-month period of data collection pre-intervention implementation<br><br>Early-post intervention: 2-month period of data collection post-intervention implementation	Sales data.<br><br>Products selected for analysis included healthy food choices confectionary, chocolate, crisps, chewing gum and other snacks.

	1 hospital convenience store		2. Products that did not met national nutritional guidelines were removed and replaced with healthier alternatives.<br>3. Removal of in-store promotion of unhealthy foods.<br>4. Incentivising healthy behaviour through altering traditional meal deal of sandwich, drink and crisps to sandwich, water and a free piece of fruit.<br>5. Healthy items like fruit were moved onto eye-level shelves and unhealthier options were placed towards the back.		Late-post intervention: 2-month period of data collection 10 months after early-post intervention period.	
**Sonnenberg et al**[37]<br><br>**USA**	Quantitative descriptive<br><br>12 wks<br><br>1 hospital cafeteria	Baseline: 166 employees<br><br>Intervention 223 employees	Traffic light labelling – Foods and Beverages classified as 'green', 'amber' and 'red' using a state government framework.<br><br>Signs describing the colours of labels were dispersed throughout cafeteria.	Primary: Mean proportion of 'green', 'amber' and 'red' purchases at baseline and full-intervention.	Not available	Surveys<br><br>Cash-register data linked to survey respondents
**Vanderlee et al**[30]<br><br>**Canada**	Cross-sectional<br><br>5 wks	Intervention group: 497 employees<br><br>Control group:	Multi-component intervention testing effectiveness of nutrition information on noticing and food consumption.	**Primary**: Influence of nutrition information on food choice	Full-intervention: 5-week data collection during full-intervention period.	Interviews

	2 hospital cafeterias	506 employees	Interventions implemented at intervention site:<br>1. Introduction of 5 digital menu boards featuring dietary energy content, sodium, saturated and total fat of food items<br>2. Healthy logo for items meeting nutritional standards<br>3. highlighting healthier menu items displayed on menu at cafeteria entrance<br>4. Intervention cafeteria reformulated recipes for some food items and removed deep fryer.<br><br>Control site: limited nutrition labelling and nutrition information available.	Nutritional composition of purchased meals		
**Van Kleef et al**[38]<br><br>**Netherlands**	Field study<br><br>4 wks<br><br>1 hospital cafeteria	Not available	Choice architecture and price-reduction.<br><br>For choice architecture:<br>Food items were displayed as either:<br>1. 25% Healthy on top, 75% unhealthy on the bottom<br>2. 75% unhealthy on top, 25%	**Primary**: Total number of snacks sold and visitors for the day.	Full-intervention: 4-week data collection during full-intervention period	Sales data obtained through counting the number of snacks left each day.

			healthy at the bottom<br>3. 75% healthy on top, 25% unhealthy on the bottom<br>4. 75% healthy on the bottom, 25% unhealthy at the top.<br><br>For price reduction:<br>All products sold for 0.85 Euros except for fresh fruit (0.50 Euros).			
**Warsaw & Morales**[40]<br><br>**USA**	Quantitative descriptive<br><br>3 yrs<br><br>1 hospital cafeteria	Not available	Permeant price changes on cafeteria menu items. Intervention phases included:<br>1. Baseline<br>2. Reduction of salad and bottled water price<br>3. Increase in cheeseburger price	Purchase of targeted food and beverage items	Baseline: 11 bi-weekly periods (every two weeks) of data collection pre-intervention implementation<br><br>Partial intervention: 22 biweekly periods of data collection during salad bar price reduction<br><br>Post-intervention: 30 biweekly periods of data collection post cheeseburger price increase	Point of sales data prices, revenue and quantities of products sold.

Study	Intervention type	Measures		Reported effectiveness	Evidence of reported effectiveness
		**Eating patterns**	**Food purchasing behaviour**		
Allan et al[23]	point-of-purchase prompt (health sign)	N	Y	Y	Reduction of energy content of products purchased/day (compared to baseline): Calories (p<0.001).
Lowe et al[24]	point-of-purchase prompt (nutrition information)<br>Price modification<br>Recipe modification	Y	Y	P	Reduction of energy content of lunch purchases by both environmental change group (EC) and environmental change plus pricing incentives group (EC-plus) compared to baseline (p<0.001).<br>EC-plus group increased fruit intake (p<0.05)<br>EC group decreased fruit intake (p<0.05)<br>Both groups reduced meat intake (p<0.05)<br>Hospital A reduced fat and sweets intake<br>Hospital B increased fat and sweet intake (p=0.005).
Stities et al[25]	point-of-purchase prompt (nutrition information)<br>Price modification	N	Y	Y	**Compared to delayed treatment group, lunch purchases from treatment group had:**<br>Less dietary energy content (p=0.01)<br>Less fat (p=0.005).<br>Compared to the full-intervention, lunch purchases during the partial intervention had:<br>Less dietary energy content (p<0.001)<br>Less fat (p<0.001).<br>**Compared to baseline, lunch purchases during partial intervention (no incentive) had:**<br>Less dietary energy content (p<0.001)<br>Less fat (<0.001).
Block et	point-of-purchase	N	Y	P	**Adjusted % change in beverage sales from baseline -**

al[26]	prompt (health message)<br>Price modification				**Price increase phase -**<br>Decline in regular soft drink sales (p<0.001)<br>Increase in diet soft drink sales (p<0.01)<br>Statistically insignificant change in zero-calorie water purchase.<br>**Education phase -**<br>Decline in regular soft drink sales (p<0.05)<br>Statistically insignificant change on purchases of zero-calorie water and diet soft drinks.<br>**Combination phase -**<br>Decline in regular soft drink sales (<0.001)<br>Increase in diet soft drink sales (p<0.05)<br>Statistically insignificant change in zero-calorie water purchase.
Geaney et al[27]	point-of-purchase prompt (nutrition information)<br>Recipe modification<br>Removal of unhealthy foods<br>Change in cooking processes	Y	N	Y	**Intervention group -**<br>Significantly lower intake of total sugars (p<0.001), total fat (p<0.000) and salt (p < 0.046) compared with non-intervention site.<br>Mean intake of salt (5.6g/d) compared to the non-intervention site (6.7g/d) did not exceed national recommendations (6g/d).<br>63% reported rarely/never using salt while cooking compared to 45% in the non-intervention site.<br>Non-intervention site participants more likely to reach the RDA for calcium (p<0.001) and consumed more iron than intervention site (not significant).
LaCaille et al[28]	point-of-purchase prompt (health message, traffic light labels, nutrition information)<br>Choice architecture<br>Recipe modification	Y	N	P	**Compared to baseline -**<br>**Intervention group -**<br>Reduced fruit intake (p=0.007) and fibre intake at 12-months (p=0.01)<br>Reduction in all high-fat/sugar foods at 12-months (p=0.02 to p<0.0001).
Lassen et al[29]	point-of-purchase prompt (health labels)	Y	N	P	**Reduction of food constituents at follow-up at intervention site (compared to baseline) -**<br>Energy per meal (p=0.030)<br>Fat % from energy (p<0.001)

					Fruit and vegetables g/100g (p=0.002)<br>Salt g/100g (p<0.001)<br>Refined sugar g/100g (p<0.009)<br>Statistically insignificant effect on wholegrains.<br>**Changes within control site at follow-up compared to baseline -**<br>Increase in energy per meal (p=0.005)<br>Increase in fat % from energy (p=0.029)<br>Insignificant change in fruit and vegetable, salt, refined sugar and wholegrain intake.
Mazza et al[34]	point-of-purchase prompt (health message, traffic-light labels, health labels)<br>Price modification<br>Increasing healthy food availability	N	Y	P	**Healthy beverage purchases -**<br>Increased during traffic-light labelling phase compared to price increase phase for soft drinks (p<0.0001)<br>Reduced during social norms message phase (p<0.01)<br>Reduced during healthy substitute phase (p=0.01)<br>Reduced during grouping healthy items phase (p<0.0001)<br>**Healthy chip purchases -**<br>Increased during traffic-light labelling phase compared to price increase phase (p=0.001).<br>Decreased during water price decrease phase (p=0.003)<br>Increased during second health message (p=0.004).
Vanderlee et al[30]	point-of-purchase prompt (nutrition information, health labels)<br>Recipe modification	**N**	**Y**	**Y**	**Compared to control site, Intervention site consumed -**<br>**Foods with -**<br>Less dietary energy (p<0.001), sodium (p<0.001)<br>saturated fat (p<0.001) and total fat (p<0.001).<br>**Beverages with -**<br>Less sodium (p=0.011), saturated fat (p=0.005) and total fat (p=0.004).<br>No statistically significant difference in dietary energy content of beverages consumed.<br>Intervention site more influenced by menu labelling (p<0.001) with 35.5% influenced by labelling claimed purchasing foods with less sodium. 30.6% claimed purchasing foods with less total energy (5.7% of entire sample).
Blake et	point-of-purchase	N	Y	Y	**Customer beverage purchases -**

al[42]	prompt (traffic-light labels)<br>Price modification				27.6% reduction in 'red' beverage purchases (95% CI= -32.2 to -23.0)<br>26.7% reduction in 'amber' beverage purchases (95% CI= -39.3 to -16.0)<br>26.9% increase in 'green' beverage purchases (95% CI= +14.1 to +39.7)<br>30% customers changed purchase due to price differential.
Dawson et al[41]	point-of-purchase prompt (health message)<br>Nutrition information<br>Menu modification<br>Increased availability of healthy foods<br>Restriction on unhealthy foods	Y	Y	Y	**After intervention implementation –**<br>42% reported eating more wholegrains<br>30% reported requesting condiments on the side<br>29% reported eating more vegetables<br>24% reported consuming more low-fat dairy products<br>17% reported eating more fruit
Dorresteijn et al[31]	point-of-purchase prompt (health message)<br>Choice architecture<br>Recipe modification	N	Y	P	**Compared to baseline -**<br>Insignificant change in reduced salt soup and lean croissant purchase during intervention period.<br>Increase in purchase of lean croissants during after-intervention period (p=0.002).<br>Increase in butter purchase during placement reversal period (p<0.01).
Elbel et al[32]	point-of-purchase prompt (Health label)<br>Price modification	N	Y	P	**Highlighting-only phase -**<br>Increase in healthier item purchase (p=0.02)<br>Reduction in dietary energy content of foods purchased (0.02)<br>Reduction in sugar purchased (p<0.032)<br>Statistically insignificant effect on total fat and sodium purchased.<br>Statistically insignificant effect on choosing fewer less-healthy foods and healthier beverages.<br>**Price modification phase –**<br>Reduction in purchase of less-healthy foods (p<0.001) and increase in purchase of more healthy beverages (p=0.001).<br>13.91 reduction in dietary energy content of foods purchased (p<0.001).<br>1.97g reduction in sugar purchased (p<0.001).

					Statistically insignificant change on total fat and sodium purchased.
Levy et al[33]	point-of-purchase prompt (traffic-light labels)<br>Choice architecture	Y	N	Y	**Labelling phase -**<br>Relative % change from baseline:<br>11.2% reduction in ‘red’ item purchases (p<0.05)<br>6.6% increase in ‘green’ item purchases (p<0.05)<br>**Choice architecture -**<br>Relative % change from labelling phase:<br>4.1% reduction in ‘red’ item purchases (p<0.05)<br>1.9% increase in ‘green; item purchases (p<0.05)
Patsch et al[35]	point-of-purchase prompt (health message, health labels)<br>Price modification<br>Increasing healthy food availability	N	Y	P	**Sales of healthy, BB burgers compared to baseline -**<br>Increased at PH (p<0.001)<br>Increased at SFMC (p<0.001)<br>Traditional burger sales decreased at both sites, however remained the majority of sales during the intervention (p<0.001).<br>**Sales of healthy, BB salads compared to baseline -**<br>Decreased at PH (p=0.304).<br>Increased at SFMC (p=0.002).<br>Traditional salad sales decreased at PH (p=0.238) and SFMC (No p-value available).
Sato et al[39]	point-of-purchase prompt (nutrition information, health labels)<br>Price modification<br>Recipe modification	N	Y	N	**Sales of healthy, HP entrees -**<br>Statistically insignificant increase from 41.8% (baseline) to 42.5% (8th intervention week)<br>**Sales of regular, unmodified entrees -**<br>Statistically insignificant decrease from 58.2% to 57.5% (8th intervention week)<br>50% customers purchasing entrees influenced by labels.
Simpson et al[36]	Choice architecture<br>Increasing healthy food availability<br>Removal/restriction of unhealthy food	N	Y	N	Fruit sales increased from 40 to over 900 units per week.<br>Statistically insignificant increase in proportion of healthy item sales.<br>Statistically insignificant reduction in food item sales (pre compared to late post-intervention):<br>Packets of sweets and chocolate (12% to 7%)<br>Children’s confectionary (3% to 2%)<br>Other snacking (8% to 7%)

					Statistically insignificant increase in food item sales (pre compared to late post-intervention):<br>Crisps (23% to 24%)<br>Blocks and bars of chocolate remained unchanged in late post intervention period.
Sonnenberg et al[37]	point-of-purchase prompt (traffic-light labels)	N	Y	Y	**Compared to baseline -**<br>Increase of food and nutrition as a factor influencing food and beverage choice from 46% to 61% after traffic-light labelling intervention (p=0.004)<br>Increase in proportion of respondents looking at nutrition information during traffic-light labelling intervention (p<0.001).
van Kleef et al[38]	Choice architecture	Y	N	P	Snack display manipulations did not impact sales of unhealthy snacks.<br>75% healthy vs 25% unhealthy assortment increased daily healthy snack sales when compared to 25% healthy vs 75% unhealthy assortment (p=0.01).<br>No interaction effect of shelf arrangement and assortment structure observed (p=0.35).
Warsaw & Morales[40]	Price modification	**N**	**Y**	**P**	**After price reduction of salads and water –**<br>Increase in biweekly salad bar sales (p=0.05) and again (p=0.001) after introduction of cheeseburger price increase (p=0.001).<br>Biweekly cheeseburgers sales decreased from $3,787 to $3,458 when salad bar price decreased. Increase cheeseburger sales after introduction of cheeseburger price increase (p=0.05).<br>Bottled water revenue decreased when price decreased (p=0.01).

Y= Yes, N= Not effective, P= partially effective.
